# Effects of a partner’s tap intervals on an individual’s timing control increase in slow-tempo dyad synchronisation using finger-tapping

**DOI:** 10.1038/s41598-020-65033-w

**Published:** 2020-05-19

**Authors:** Kazuto Kimura, Taiki Ogata, Yoshihiro Miyake

**Affiliations:** Department of Computer Science, Tokyo Institute of Technology, Yokohama, 266-8502 Japan

**Keywords:** Sensorimotor processing, Social behaviour, Human behaviour

## Abstract

In musical ensembles, musicians synchronise their movements with other members of the ensemble at various tempos. This study aims to investigate the extent of tempo dependency of own and partner’s timing information on rhythm production. We conducted a dyad synchronisation-continuous finger-tapping task. First, two participants synchronised with the same auditory metronome at various tempos. Subsequently, after stopping the metronome, the participants maintained the tempo with the presentation of the partner’s tap timing via auditory signals. This task was conducted in six metronome tempo conditions at 700 to 3,200 ms in 500 ms step. It was found that the partner’s previous inter-tap intervals increased as the metronome tempo decreased. The effects of own previous inter-tap intervals and synchronisation errors between own and the partner’s tap timing did not depend on the metronome tempo. Therefore, timing control in dyad synchronisation was affected by the partner’s tempo more strongly in slow than fast tempos. This strong effect of the partner in slow-tempo rhythm synchronisation could be due to stronger attention to the partner’s movement timing in slower tempos than in fast tempos.

## Introduction

People are capable of co-ordinating rhythms with others as observed in musical ensembles like orchestras. Musicians synchronise the timing of their movements in musical ensembles with varying tempos. The tempo of the music appears to particularly cause difficulties for musicians attempting to synchronise with other players while maintaining the appropriate tempo. Merker conjectured that the capability to synchronise with others within a wide range of tempos may be specific to humans and possibly played a crucial role in the development of music and language^1^. Desain and Honing demonstrated that the correlation of log-transformed normalised rhythm between tempo conditions in music is smaller than that within a single tempo condition, using an experiment in which a participant played a piano in several tempo conditions^2^. This raises the following question: how does tempo dependency in rhythm production compare between subjects or individuals?

The fundamental features and mechanisms of individual rhythm production have been investigated using a finger-tapping task^3–9^ with and without external stimuli (e.g. constant-tempo metronome). In this task, participants tap their finger to synchronise with an external stimulus (synchronisation task) or to maintain a tempo presented in advance (continuous task). Previous studies have found that people control their tap timing by using their prior personal tap timing and the external stimuli timing during a synchronisation task: i.e. previous personal inter-tap interval (ITI), previous inter-stimuli onset interval (IOI), and a previous synchronisation error (SE) between their own tapping and the stimulus^10–16^. Previous studies have also found that people control their tap using their prior personal ITI in a continuous task without external stimuli.

First, it is well established that the length of an individual’s prior personal ITI will impact the length of that individual’s subsequent personal ITI. Wing and Kristofferson investigated the temporal dependency of ITIs on prior ITIs using a synchronisation-continuous tapping task (SC tapping task)^17,18^. In the SC tapping task, first, the participant performs the synchronisation task with an auditory constant-tempo metronome, and then the metronome stops, after which the participant aims to maintain the tempo set by the metronome. Using a fast metronome tempo (from 180 ms to 400 ms IOI), a lag 1 negative auto-correlation was found in the ITI time series of the continuous task, indicating that a shorter ITI followed a longer ITI, and longer ITI followed a shorter ITI. Individuals personal temporal dependency ITI differed by tempo (fast vs. slow). Madison conducted a SC tapping task using IOIs from a metronome (between 400 ms to 2,200 ms) and found that negative first order covariance turned positive as the tempo slowed. This result shows ITIs tended to drift—i.e. ITIs gradually tended to become longer or shorter^19^. In addition, the lag 1 negative covariance in the synchronisation task, became larger as the tempo slowed, when the IOI was >1,200 ms. This indicates that the degree of the ITI tendency to reduce after an extended ITI (and vice versa) is greater at slower tempos.

Second, the effect of IOI on timing control was explored with varying metronome IOIs. In a synchronisation task with fast metronomes (with IOIs <1,500 ms), the ITIs followed the variation of IOIs with a lag 1 delay when IOIs had sinusoidal, periodic, or random modulation^10,20–22^. Individuals tended to produce short ITIs when previous IOIs were short and long ITIs when previous IOIs were long. Synchronisation tapping with predictable IOIs patterns (e.g. sinusoidal) or patterns that resemble typical music patterns were also conducted^22,23^. When the IOIs pattern standard deviation was too little or far from the typical pattern of predicting change in IOIs, ITIs followed previous IOIs. When the IOI followed a sinusoidal pattern with a large enough amplitude or resembled typical music patterns, the variation in ITIs and IOIs synchronised—i.e. ITIs and IOIs positively correlated at lag 0. This indicated that people predictably controlled own tap timing when the timing modulation of external stimuli was predictable. In the synchronisation task using slow metronomes with constant IOIs (>1,800 ms), timing control differed on the synchronisation task using a fast tempo metronome^24^. Miyake *et al*. conducted a dual-task study in which participants simultaneously performed a synchronisation task with constant-tempo metronome and a word memory task. In the latter, the participants were asked to remember 4–5 words displayed on the monitor. When the metronome IOIs were 1,800–3,600 ms, compared to single synchronisation tasks, the dual task increased the rates of reactive tapping, in which the participants’ tap occurred after exposure to the corresponding metronome stimuli. However, when the metronome IOIs were set at 450–1,500 ms, such differences between single and dual tasks were not observed. These results suggest that the strategy of timing control in synchronisation using a slow-tempo stimulus differs from that in the case of a fast-tempo stimulus.

Finally, the effect of SEs on timing control during a synchronisation task was investigated in the context of phase correction, which is the tendency to adapt subsequent taps to make the next SE shorter than the previous SE^21^. People tend to produce a shorter subsequent ITI or tap early when the previous tap was later than the corresponding stimuli onset, while they produce longer subsequent ITI or tap slowly when the previous tap was earlier than the corresponding stimuli. The degree of this phase correction was investigated using the synchronisation task with a metronome including a sudden change in stimuli onset timing (i.e. local perturbation)^23,25–31^. The degree of phase correction depends on the metronome tempo^32,33^ and increases as the tempo slows and when the IOI is between 400–1,200 ms^33^.

Recent studies have found that timing control in synchronisation with other people has different characteristics from synchronisation with the metronome^34–39^. Konvalinka *et al*. conducted a dyad SC task^34^, wherein two participants simultaneously synchronised an isochronous metronome using finger tapping and maintained the tempo after the metronome stopped. In the continuous task, the two participants perceived own or partner’s tap timing via auditory stimuli. The authors used three conditions for the metronome tempo: 400, 500, and 625 ms. When both the participants perceived their partner’s tap timing, lag 1 and −1 cross-correlations were detected between own and partner’s ITIs in all the conditions. This result indicated that the participants followed their partner’s prior ITIs. Okano *et al*. also investigated timing control in synchronisation between two people using the dyad SC task^39^. The participants perceived the partner’s tap timing via auditory signals to each other. The authors used three metronome tempo conditions: 300, 500, and 800 ms. This was analysed using the regression model setting *δ*ITI (fluctuation of ITI from previous ITI) as a dependent variable and a previous *δ*ITI (difference of previous own ITI and partner’s ITI) and the multiple of the two as the explanatory variables. The result showed that the faster the metronome tempo, the less the difference between own and partner’s ITI affected the next *δ*ITI.

These previous studies revealed unique characteristics in dyad synchronisation. However, these studies used relatively early tempo, under-1,000 ms tempo, for the target tempo in the dyad synchronisation task. In solo-tapping tasks with or without metronome, the target tempo was found to influence the participant’s timing control, as mentioned above^19,24,32,33^. Thus, timing control in dyad synchronisation would be affected by the target tempo. In addition, the effects of own and partner’s ITIs, and the SEs on timing control in the synchronisation task have been investigated individually. However, their effects were found to be distinct and should thus be investigated simultaneously. This study aims to simultaneously reveal the impact of personal and partner’s ITI, and the SE between own and partner’s tap timing, on timing control in a dyad synchronisation task at a slow tempo.

For this purpose, we conducted a dyad SC tapping task using a metronome with IOIs between 700–3,200 ms in steps of 500 ms. At the beginning of each trial, 10 metronome stimuli are simultaneously presented to two participants. From the fourth metronome stimulus, the participants start to tap their right index finger to synchronise with the metronome. After the metronome stops, the participants continue tapping to sustain the tempo set by the metronome. In this continuous task, the participants are presented with their partner’s tap timing via auditory signals and instructed to synchronise their tap timing with their partner’s tap. However, it is usually difficult to simultaneously maintain the metronome tempo and synchronise with a partner, and hence, we instructed the participants to prioritise maintaining the metronome tempo. To simultaneously investigate the effect of previous own and partner’s ITIs and SEs on timing control, we estimated the degree of their effect on the subsequent *δ*ITIs using a multiple regression analysis.

## Results

### Mean tempo and SEs

**Figure 1:**
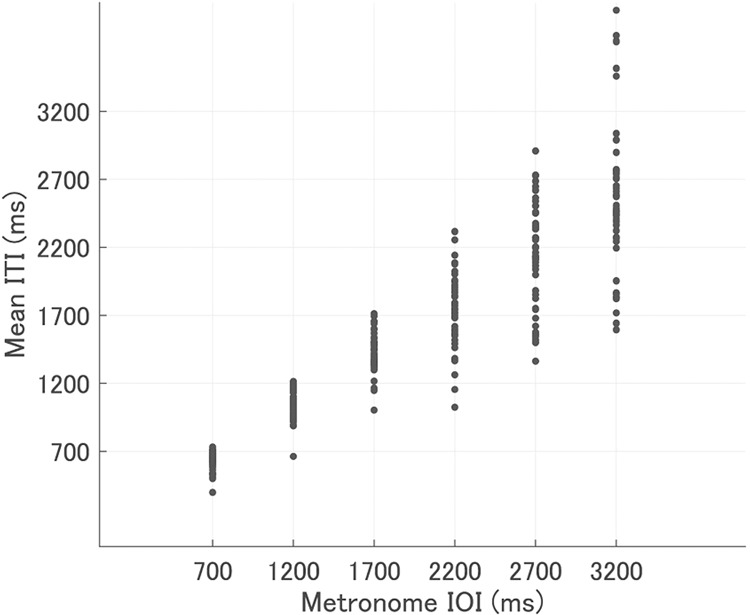
Scatter plot between the metronome IOIs and mean ITIs. The x-axis shows the metronome IOIs and the y-axis shows the mean ITIs for each trial. A dot means a data point in each trial.

Okano *et al*. showed that ITIs gradually decreased in a dyad SC tapping task using a metronome with 300, 500, and 800 ms tempos^39^. Thus, we calculated the mean ITIs in each trial to investigate whether the mean ITIs change when the metronome tempo shifts to slower conditions. Figure 1 shows a scatter plot of the metronome IOIs and mean ITIs of the last 40 taps from the 21st to the 60th tap, after the metronome stops, in each trial. The mean ITIs also tended to decrease compared to the baseline metronome tempo. In addition, the degree of ITI decrease was different between the trials even in the same metronome tempo conditions. Therefore, we compared the effects of own and partner’s ITIs and SEs on the subsequent ITIs based on the mean ITIs for each trial.

**Figure 2:**
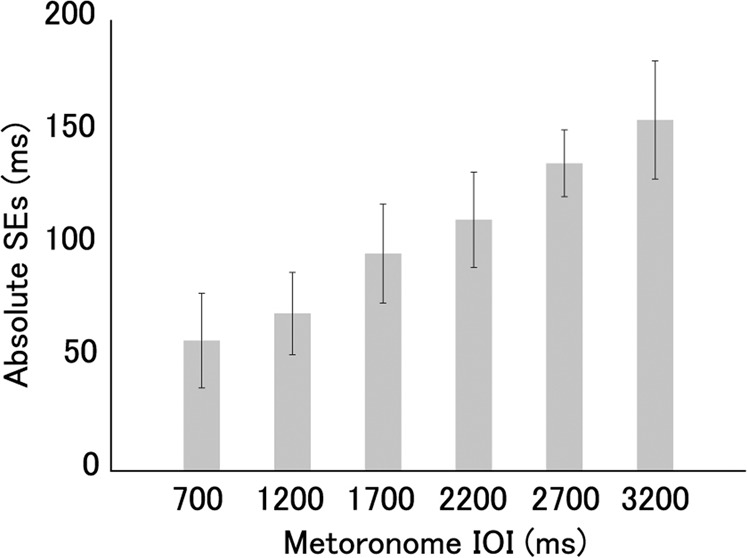
Averaged SEs for each tempo condition. The error bars depict standard deviations between the participants. We found significant differences between all pairs of the tempo conditions (*p* < 0.001).

SEs increased as the metronome tempo increased. The average absolute SE at each tempo condition is shown in Fig. 2. An ANOVA for SEs using tempo condition as the factor revealed significant differences (*F*(3.16, 72.61) = 208.40, *p* < 0.001). Multiple comparison showed significant increases of SEs from 700 ms to 1,200, 1,700, 2,200, 2,700, and 3,200 ms conditions (*t*(23) = 4.89, *p* < 0.001; *t*(23) = 10.41, *p* < 0.001; *t*(23) = 25.56, *p* < 0.001; *t*(23) = 21.71, *p* < 0.001; *t*(23) = 20.09, *p* < 0.001, respectively), from 1,200 ms to 1,700, 2,200, 2,700, and 3,200 ms conditions (*t*(23) = 6.86, *p* < 0.001; *t*(23) = 13.59, *p* < 0.001; *t*(23) = 22.01, *p* < 0.001; *t*(23) = 19.01, *p* < 0.001, respectively), from 1,700 ms to 2,200, 2,700, and 3,200 ms conditions (*t*(23) = 4.34, *p* < 0.001; *t*(23) = 10.72, *p* < 0.001; *t*(23) = 13.48, *p* < 0.001, respectively), from 2,200 ms to 2,700 and 3,200 ms conditions (*t*(23) = 6.93, *p* < 0.001; *t*(23) = 9.28, *p* < 0.001, respectively), and from 2,700 ms to 3,200 ms conditions (*t*(23) = 5.52, *p* < 0.001).

### Tempo dependency of the effects of own and partner’s ITIs and SEs on subsequent ITIs

To investigate the effects of own and partner’s tap timing on timing control, we used a multiple regression analysis while setting *δ*ITI as a dependent variable and previous own and partner’s ITIs, and the previous SEs as explanatory variables, as observed in the following equation.

$$\delta IT{I}_{i}(n)=\alpha IT{I}_{i}(n-1)+\beta IT{I}_{j}(n-1)+\gamma S{E}_{ij}(n-1),$$

where *α*, *β*, and *γ* are standard multiple regression coefficients and indicate the degrees of previous own ITIs, partner’s previous ITIs, and previous SEs, respectively. The *α*, *β*, and *γ* were calculated for each participant in each trial. Before calculating these coefficients, to equalise the effect of the variable scales between trials, we standardised the distributions of the ITIs and SEs to a normal distribution with a mean of 0 and a standard deviation of 1. Then, we calculated the correlation coefficients between standard partial multiple coefficients and ITIs’ means. Table 1 shows the results of the correlation analysis between the regression coefficients and the mean ITIs for each trial.

**Table 1:** Results of the correlation analysis between the standard partial multiple regression coefficients—*α*, *β*, *γ*—and the mean ITIs.

Standard partial multiple regression coefficient	Correlation coefficient r	*p* value
*α*	−0.014	0.734
*β*	0.238	<0.001
*γ*	0.021	0.615

These coefficients, *α*, *β*, *γ*, were from the timing control model: $$\delta IT{I}_{i}(n)=\alpha IT{I}_{i}(n-1)+$$$$\beta IT{I}_{j}(n-1)+\gamma S{E}_{ij}(n-1)$$; and *α*, *β*, and *γ* meant the effect of own ITI, partner’s ITI, and SE on the next *δ*ITI. Mean ITIs were the index of tempo.

**Figure 3:**
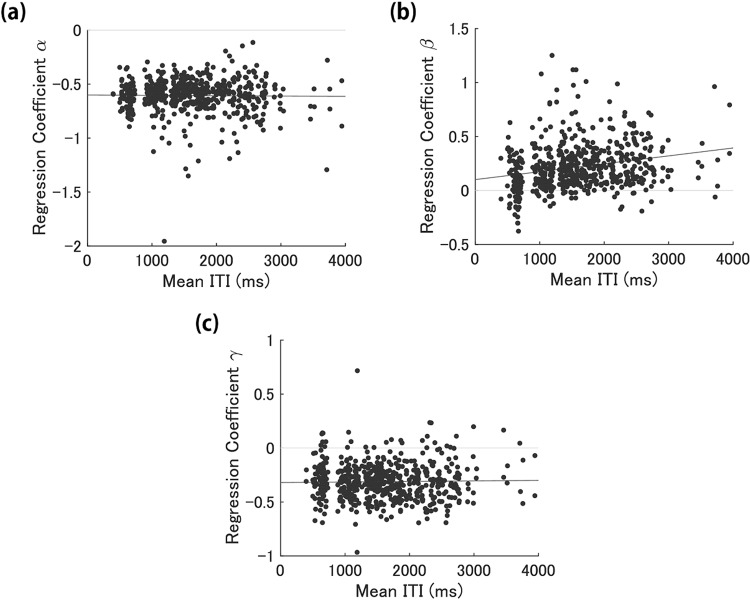
Scatter plot between the degree of the regression coefficient (**a**) *α*, (**b**) *β*, (**c**) *γ* and mean ITIs. The x-axis shows mean ITIs and the y-axis shows the regression coefficient *α*, *β*, and *γ*. A dot indicates a participant’s data point in one trial. A line is the regression line of the regression coefficient *α*, *β*, and *γ* as a function of the mean ITIs and calculated with polyfit function in MATLAB.

Figure 3(a) shows a scatter plot of *α* and the mean ITIs. In most trials, the *α* value was negative and in between −1.0 and −0.4. The correlation coefficient between *α* and the mean ITIs was −0.014. There was no significant correlation found (*p* = 0.734). This result revealed that the degree of the effect of own ITI on the next ITIs was independent from the mean tempo. Figure 3(b) shows a scatter plot of *β* and the mean ITIs. In most trials, the *β* value was in the range of −0.2 to 0.6. The correlation coefficient between *β* and the mean ITIs was 0.238. There was a significant correlation *p* < 0.001). Therefore, the degree of the effect of the partner’s ITI on the next ITIs increased when the mean tempo became slow. Figure 3(c) shows a scatter plot of *γ* and the mean ITIs. In most trials, the *γ* value was negative and within the range of −0.6 to −0.1. The correlation coefficient was 0.021. There was no significant correlation between *γ* and the mean ITIs (*p* = 0.615). Thus, the degree of the effect of SEs on the next ITIs was independent from the mean tempo. To investigate whether the models of each trial fit to the data, we conducted ANOVA for *R*^2^ using the explanatory variables as the factors. The results showed that the *p* values were below the significance level (0.05) for the explanatory variables in all trials.

## Discussion

The purpose of this study was to investigate how the effects of own and partner’s tap timing on timing control are affected by the mean tempo in synchronisation with the partner. We conducted a dyad SC tapping task with several metronome IOIs as target tempos. We estimated the degree of the effects of own previous ITIs, partner’s previous ITIs, and SEs on the next *δ*ITIs using regression analysis. As a result, we found that the effect of the partner’s ITI on the next *δ*ITI became strong as mean ITIs increased, but the effects of own ITIs and SEs did not. The statistical test for *R*^2^ showed good fit of the models.

The *α* value (Fig. 3(a)), which shows the degree of the effect of own ITI on *δ*ITI, was negative and in the range of −1.0 to −0.4 in all metronome tempo conditions in our experiment. This result indicates that a short ITI followed a long ITI and a long ITI followed a short ITI in the dyad SC tapping task with fast and slow tempos. In the solo continuous task with faster metronome tempos (under 700 ms), the same temporal dependency of own ITIs was found but not with slower tempos^19^. This is because the ITI drift was strong in the solo slow tempo continuous task and covered the temporal dependency between own ITIs. We did not find the effect of the mean ITIs on the *α* value in the dyad SC tapping task. This suggests that the ITI drift in the slow-tempo dyad SC task did not exist or was not strong compared to the slow-tempo solo continuous task.

More information could be obtained from our analysis of the *β* value (Fig. 3(b)); the longer the mean tempo was, the larger the degree of the effect of partner’s ITIs on the next own ITIs. This result revealed that in slow-tempo synchronisation with a partner, timing control was affected by the partner more strongly than in fast-tempo synchronisation. Okano *et al*. showed that the difference between own and a corresponding partner’s ITIs increases as the metronome tempo becomes slower^39^. The results of this study suggest that the tempo dependency of the effect of ITI difference is due to the dependency of the effect of partner’s ITI, not those of the effect of own ITI. A previous study with a solo synchronisation task including a dual task suggested that timing control of tapping with slower tempo is intentional and requires attentional resources^24^. People tend to tap earlier than the stimuli in a synchronisation task with a metronome^4,6^. This tendency, which is called negative mean asynchrony (NMA), suggests that people tap with anticipatory timing control. NMA turns into reactive tapping in synchronisation tapping with slower tempo over 1,800 ms and the ward memory task at the same time. In other words, participants tend to tap later than stimuli when faced with a dual task and slow tempo. This suggests that participants use resource and control timing in an anticipatory synchronisation tapping task with slower tempo metronomes. This study showed that the degree of the effect of partner’s ITI on timing control became larger as the tempo became slower. The results suggest that participants possibly used resources to follow the partners’ ITIs.

The *γ* value (Fig. 3(c)), which shows the degree of the effect of SE on timing control, was negative and fell within the range of −0.8 to −0.1 among all tempos in this experiment. This result indicates the tendency to adopt a longer subsequent ITI when a participant taps earlier than the other’s tap as per stimuli and a shorter subsequent ITI when the participant taps slower than the other. In addition, the effect of SEs on timing control was independent from the mean tempo much like that of own ITIs. In a previous study using a synchronisation finger-tapping task with a constant-tempo metronome or with adaptive timing modulation stimuli, people increased the ITI when the participant tapped earlier than the stimuli, and vice versa^32,33^. This study also estimated the degree of this timing correction. The estimation showed that the degree of tap correction became larger as the tempo slowed. Therefore, the dependency of phase correction degree on the tempo in dyad synchronisation would be likely different from the synchronisation with a constant tempo metronome and adaptive stimuli. However, because the phase correction in the previous study and the effect of SEs on *δ*ITIs in our study are not the same, these results are not comparable.

The mean ITIs were much faster in slower tempo conditions than in faster tempo conditions (Fig. 1). This is due to the tempo dependency of the effect of partner’s ITI and independency of own ITIs. As mentioned above, the participants controlled their tap timing to track the partner’s previous ITI. In addition, it is known that people perceive shorter intervals of continuous external stimuli than the actual one^40^. Therefore, the mean ITIs of participants tended to decrease in all the tempo conditions. Meanwhile, the participants tapped early or late when their previous ITI was long or short, respectively. In this manner, the participants tried to keep their tempo constant. In the slower tempo conditions, the effect of the partner’s ITIs on timing control was stronger than in the faster tempo conditions, but the effect of own ITIs was not. Thus, the ITIs decreased more in the slower conditions due to the relatively strong effect of the partner’s ITIs. Another possibility of large acceleration of the tempo in the slower tempo conditions is due to the larger absolute SEs in the slower tempo conditions than in the faster conditions (Fig. 2). During synchronisation tapping with isochronous sequence, people tend to tap earlier (later) if the previous stimuli is earlier (later) than the own previous tap^8,9^. In addition, the effect of this earlier stimuli on tap timing is larger than that of the later stimuli^41^. Thus, in our experiment, the tap timing was affected more strongly by the partner’s earlier timing than the later one, and the ITIs decreased in many trials. Gibbon *et al*. proposed a model for perception of interval timing^42^, which model provides large SEs in slow tempo condition. In this model, timing or interval is represented relatively, not absolutely. Based on this model, long intervals cause large variability of interval perception and production. This large variability would lead to large SEs in slow-tempo synchronisation.

At the 2,200, 2,700, and 3,200 ms conditions, the decrease in the mean ITIs was larger than in the other conditions. The mean ITIs were approximately half of the metronome tempo in some trials because of the upper limitation of “feeling of nowness”^43^. The average limitation in which people can perceive the continuous stimuli as a whole or a subjective temporal *gestalt* is from 2 to 3 s^44–50^. Therefore, at over 2,200 ms conditions, the tempo of the metronome would be above this limitation for some participants. This would make it difficult for the participants to perceive the partner’s sequence as a whole and/or to tap their finger rhythmically with the metronome tempo. Thus, the mean ITIs would move below the limitation of “feeling of nowness.”

In some trials, the mean ITIs increased compared to the metronome’s tempo (Fig. 1). Of all 288 trials, the mean ITI was longer than the IOI in 21. In these trials, the participants might have intentionally tapped late or increased their ITIs to prevent tempo acceleration. If both the participants tap late, the ITIs would increase through mutual tracking of the partner’s ITI. This should be investigated in future studies.

In conclusion, our purpose was to reveal the effect of tempo on timing control in synchronisation with other persons. To examine this, we conducted a dyad SC tapping task with metronome tempo in the range of 700–3,200 ms. We found a positive correlation between the effect of partner’s ITIs on the subsequent tap and the mean tempo. However, there was no significant correlation between the effect of own ITI or SE and mean tempo. The results suggest that participants tend to rely on partner’s ITI more when the mean tempo is slower.

## Methods

### Participants

A total of 24 participants (4 women and 20 men; 21–31 years old) were sampled in this experiment and were divided into 12 pairs (all consisting of same sex participants). All participants were right-handed. No participant reported auditory and movement impairments. All participants reported no experience in professional music training. This experiment was conducted in accordance with the Declaration of Helsinki and approved by the Research Ethics Review Committee of the Tokyo Institute of Technology. We obtained informed consent from participants in writing.

### Material and stimuli

Pressure sensors (FSR-406, Interlink Electronics, US) were used for recording the timing of participants’ taps. Auditory stimuli were sent to participants via earphones (SHE3010WT, PHILIPS, Nederland). Auditory stimuli from the metronome and stimuli from the partner were rectangle-wave, 500 Hz pure tones, with a 100 ms duration. White noise was sent to participants from a personal computer (Latitude 7280, DELL, US), via headphones (HPH-50B, YAMAHA, Japan) to prevent the effects of outside noise. Arduino (Arduino Mega2560 Rev3, Arduino, US) was used for controlling stimuli and recording tap timing. The temporal resolution to control stimuli and record tap timing was 1,000 Hz.

### Task and condition

The task was a dyadic SC tapping task consisting of synchronisation and continuous phases. First, 10 isochronous metronome stimuli were presented to each participant simultaneously. Then, pairs of participants began tapping using their index finger from the presentation of the fourth stimulus to synchronise with the metronome. In this synchronisation phase, the participants did not perceive the partner’s tap timing. After the metronome stopped, the participants continued tapping while maintaining the metronome’s tempo. In this continuous phase, the timing of a participant’s taps was sent to the partner as auditory stimuli.

To investigate the tempo dependency on timing control in dyad synchronisation, the tempo for the metronome was set at six conditions: 700, 1,200, 1,700, 2,200, 2,700, and 3,200 ms. People perceive the continuous stimuli as a whole or a subjective temporal *gestalt*^44^. The limitation of this “feeling of nowness”^43^ is known to be from 2 to 3 s on average^44–50^. In fact, in synchronisation with an isochronous metronome using finger tapping, the tapping tended to be earlier/later than the corresponding stimulus when the metronome tempo was under/over 3,600 ms^24^. This result suggests that at over 3,600 ms conditions, the participants did not synchronise to the metronome rhythmically, but relatively tapped to each stimulus. Thus, we set the maximum tempo at 3,200 ms.

### Procedure

The experiment was conducted in a soundproof room. The participants sat at desks with their backs to each other. During the task, participants wore an eye mask and were presented with white noise via headphones. The volumes of the auditory stimuli and white noise were kept constant throughout the experiment. Each trial continued until 60 taps were recorded in the continuous phase.

Each session was composed of six trials, consisting of each condition, and four sessions were conducted per pair. Thus, a total of 24 trials were conducted per pair. The order of conditions was randomised in each session and counterbalanced. Practical trials were conducted at least once for each condition. The experiment lasted approximately 4 hours, including rest time. At least a one-minute break was provided between trials and between sessions. In addition, five-minute breaks were taken at least once every 20 minutes (4–6 trials). Furthermore, the participants were allowed to take rest anytime if they wanted.

### Statistics

The participant pairs were divided into Participant A and Participant B. We defined *Tap*_*A*_(*n*) and *Tap*_*B*_(*n*) as Participant A’s and B’s n-th tap, respectively. ITI, SE, and *δ*ITI were defined as the following equations.

$$IT{I}_{i}(n)=Ta{p}_{i}(n)-Ta{p}_{i}(n-1),S{E}_{ij}(n)=Ta{p}_{i}(n)-Ta{p}_{j}(n),\delta IT{I}_{i}(n)=IT{I}_{i}(n)-IT{I}_{i}(n-1),$$

where (*i*, *j*) = (*A*, *B*) *or* (*B*, *A*).

To investigate the effects of own and partner’s tap timing on timing control, we used a multiple regression analysis and set *δ*ITI as a dependent variable and previous own and partner’s ITI, and SE as explanatory variables, as illustrated in the following equation.

$$\delta IT{I}_{i}(n)=\alpha IT{I}_{i}(n-1)+\beta IT{I}_{j}(n-1)+\gamma S{E}_{ij}(n-1),$$

where *α*, *β*, and *γ* are standard multiple regression coefficients and indicate the degree of previous own and partner’s previous ITI and previous SE, respectively. The *α*, *β*, and *γ* were calculated for each trial. Before calculating the coefficients, we standardised the distributions of ITIs and SEs to a normal distribution with a mean of 0 and a standard deviation of 1. We conducted an ANOVA for the explanatory variables as the factors to test the *R*^2^ or the model fit for the variables. We set the significance level at 0.05 and used the Holm-Bonferroni method to modify the significance level between trials. Then, we calculated the Pearson’s correlation coefficients between the standard partial multiple coefficients and the means of ITIs for each trial. We used a test for no correlation. Multiple regression, statistical test for *R*^2^, and correlational analyses were performed with MATLAB (Math Works, US). For the SEs, we conducted repeated ANOVA for the tempo conditions as factors. We used Shaffer’s method for the multiple comparison, The ANOVA and multiple comparison for SEs were conducted using R (version 3.5). We used the tapping data of the last 40 taps, from the 21st to the 60th tap after the metronome stopped. All data from the 24 trials of each participant were used in the statistical analysis (n = 576).
